# 

**Published:** 1821-05

**Authors:** 


THE LONDON

### Medical and Physical Journal.

5 OF VOL. XLV.] MAY, 1821. [NO. 267.

NOTICE.

*The Proprietors acquaint the* MEDICAL PROFESSION, *that the* GENERAL INDEX *to this Journal is published, comprising an Analytical Table of its Contents, from* VOLUME ONE TO FORTY *inclusive; carefully arranged in Alphabetical Order, with References to the whole of the cited Authorities, under their nominal Characters, &c. and forming an indispensable* APPENDIX *to the Journal; in one large* 8*vo. volume, price* 21*s.*

### MONTHLY CATALOGUE OF MEDICAL BOOKS.

A Monthly Journal of Popular Medicine: explaining the Nature, Causes, and Prevention of Disease, &c. Conducted by Charles T. Haden, Surgeon to the Chelsea and Brompton Dispensary, &c. Nos. I. and II. 1s. 6d.

A Treatise on Acupunctuation. By James Morss Churchill. 4s. boards.

A Practical Treatise on Diseases of the Heart, &c. By H. Reeder, M.D. 10s.

A Manual of the Diseases of the Human Eye. By George C. Monteath, M.D. Illustrated with Engravings. 2 vols. 8vo.

A View of the Structure, Functions, and Disorders of the Stomach and Alimentary Organs of the Human Body. By Thomas Hare, F.L.S.

A Supplement of the Pharmacopœias. By S. F. Gray. New Edition.

Practical Observations on Disorders of the Liver, &c. By Dr. Ayre. 2d Edit.

[HIGHLEY AND SON, FLEET-STREET.]

